# Evaluating the impact of clinical and translational pilot funding using multiple outcome metrics

**DOI:** 10.1017/cts.2024.686

**Published:** 2025-01-08

**Authors:** Ingrid Philibert, Angela Batson, Amanda Fletcher

**Affiliations:** 1 Great Plains IDeA-CTR, University of Nebraska Medical Center Omaha, Omaha, NE, USA; 2 Frank H Netter MD School of Medicine at Quinnipiac University, North Haven, CT, USA; 3 Parlay Consulting, Omaha, NE, USA; 4 YCCI Research Administration, Yale, New Haven, CT, USA

**Keywords:** Clinical and translational research, Translational Science Benefits Model (TSBM), idea-ctr, pilot project Program, extramural funding

## Abstract

NIH/NIGMS-funded IDeA-Clinical and Translational Research (CTR) networks seek to expand translational research infrastructure to support research that has at its endpoints measurable clinical, public health, technological, or economic benefits. This retrospective case study followed 14 projects that received Pilot funding from the Great Plains IDeA-CTR (GP IDeA-CTR) at the University of Nebraska Medical Center. It focuses on the impact of pilot funding and GP IDeA-CTR resources on subsequent clinical and translational research. Metrics include extramural awards, lessons learned that relate to clinical and translational research infrastructure, and demonstrated and potential benefits using the Translational Science Benefits Model (TSBM).

## Introduction

Institutional Development Awards for Clinical and Translational Research (IDeA-CTR) were established by the NIH National Institute for General Medical Studies (NIGMS) to advance clinical and translational research in states and regions with lower aggregate NIH funding [1]. The aim is to develop the clinical and translational research infrastructure and workforce to improve the health of individuals and populations.[2] The Great Plains IDeA-CTR (GP IDeA-CTR), headquartered at the University of Nebraska Medical Center, was established in 2016 with the aim of enhancing research capacity to improve the health of the residents of Nebraska and the Dakotas.

For this retrospective case study, the GP IDeA-CTR’s Tracking & Evaluation Core examined 14 projects that received Pilot Program (Pilot) funding between 2017 and 2019 to assess the longer-term impact of this “seed” funding. Pilot funding aims to help investigators test promising initial research projects to collect preliminary data for subsequent extramural grants. We explored how projects are related to regional health priorities and categorized projects using specific and measurable indicators in the 4 domains of Washington University’s Translational Science Benefits Model (TSBM): Clinical, Community, Economic, and Policy Benefits [3]. The TSBM is designed to show pragmatic benefits by focusing on demonstrated and potential future outcomes of clinical and translational research.

## Methods

The 14 Pilot projects were part of an initial case study of 22 studies conducted in the spring of 2021. In June 2023, we contacted the same 22 investigators for a follow-up survey. Eight did not respond or declined to participate, resulting in 14 projects in our sample. Projects were initiated between 2017 and 2019, with the majority (10) initiated in 2018.

We sought to answer three questions: 1) Whether Pilot Program-funded projects secure subsequent funding and the collective return on investment (ROI); 2) the impact or potential future impact of funded projects using a TSBM case study template [4] ; and 3) lessons learned that are relevant to enhancing the GP IDeA-CTR’s research infrastructure.

For the initial portion of the study in the spring of 2021, we collected information from NIGMS-required research reporting, to reduce the burden on participants, a brief survey pilot-funded researchers, and semi-structured Zoom-based interviews with six investigators. Interviews lasted between 30 and 40 minutes; electronic transcripts were edited by IP and another GP IDeA-CTR staff member. We adapted the TSBM case study approach [5] to create a 13-item reporting template and populated it with data from interviews, surveys, and reporting to NIGMS. We mapped pilot projects to one or more TSBM indicators. The 2023 follow-up study used required reporting data, the GP IDeA-CTR’s annual awardee survey, and a 6-item follow-up survey. The interviews and the 2021 survey collected data on how the GP IDeA-CTR had supported projects and facilitated translational science, the challenges investigators experienced, and what the administrative core could do to alleviate them. Surveys used an assent paragraph in the opening statement noting that completion of the survey served as the investigators’ consent to inclusion of their projects and data collected in our analysis and reporting. The intent of the Pilot funding is to support projects that produce preliminary data for future extramural awards, and we analyzed the 14 projects in our sample in two ways. We obtained corroboration from investigators that subsequent funding was connected to their initial projects. We consulted the Reporter.gov database to identify the amount of funding for NIH-funded projects and used the GP IDeA-CTR’s post-award database or asked investigators for subsequent funding amounts. The project was declared not to require IRB review by the University of Nebraska Medical Center’s IRB decision tool.

## Results

### Projects, Their Focal Areas, and TSBM Domains

The Pilot projects, member sites involved, and their designation as community-engaged or other clinical and translational research projects are shown in Table 1. The 14 studies represented all institutional partners of the GP IDeA-CTR for the initial funding period and included four community-engaged and ten other projects. Twelve projects targeted conditions on the GP IDeA-CTR’s Health Priorities List (HPL), with that determination made by the chair of the GP IDeA-CTR’s Community Advisory Board. Regional health priorities encompass the following: 1) behavioral health/substance abuse; 2) violence as a public health issue; 2) obesity prevention and treatment; 3) aging and age-related cognitive impairment; 4) injury prevention; 5) technologies to improve rural health; 6) clinical care and community services at schools, food banks similar sites; and 7) addressing health disparities based on social determinants of health, race, ethnicity or geography.

**Table 1.** Pilot-funded projects included in the retrospective case study

*Awardee*	*Project*	*HPL Domain(s)*	*TBSM Domain* *(demonstrated/potential)*	*Institution*
*Community-Engaged Projects*				
1. Kim, J.	Developing Strategies to Implement Evidence-Based Colorectal Cancer Screening Intervention Using a Participatory Approach	Addressing health disparities based on rural geography	Community Benefits – Community Health Services (potential) Accessibility of Care (potential) Disease Prevention and Reduction (potential)	UNMC
2. King, K.	Get Your Mind Right: Feasibility of a mental health intervention for African American fathers in North Omaha	Behavioral health AND Addressing health disparities based on race	Community Benefits – Community Health Services (potential) Accessibility of Care (potential) Disease Prevention and Reduction (potential)	UNMC
3. Lally, R.	Facilitating Oncology Patient-Clinician Communication via E-health Innovations	Behavioral health AND Addressing health disparities based on gender and rural geography	Clinical Benefits – Software Technology (potential)	UNMC
4. McFadden, L.	Dirty Little Secrets: Wastewater Epidemiology Use to Determine Community Drug Use	Behavioral health and substance abuse	Clinical Benefits – Investigative Procedures (demonstrated) Community Benefits – Public Health Practices (potential) Policy Benefits – Scientific Research Report (demonstrated)	USD
*Other Projects*				
5. Hackney, K.	Effects of Eight Weeks of Exercise Training and Time-restricted Feeding on Body Composition, Muscle Endurance, Metabolism, Cardiovascular Risk and Dietary Intake in Overweight Sedentary Males and Females	Obesity prevention and treatment	Disease Prevention and Reduction (potential)	NDSU
6. Lalonde, K.	Effects of Hearing Aid Compression on Temporal Cues in Audiovisual Speech	Aging and age-related cognitive impairment	Clin. Benefits – Diagnostic Procedures (potential) Community Benefits – Quality of Life (potential)	BNRH
7. Nelson, T.	Developing Executive Control, Obesity Risk and Behavioral Health Problems: A pilot fMRI study	Obesity prevention and treatment	Clin. Benefits – Investigative Procedures (demonstrated) Clinical Benefits – Biomedical Technology (demonstrated)	UNL
8. Schmaderer, M.	Self-Management Intervention using Mobile Health for the Multimorbid	Behavioral health AND Technologies and models to improve health access	Clin. Benefits – care delivery (potential) Clinical Benefits – Software Technology (potential) Community Benefits – Consumer Software (potential)	UNMC
9. Schwartz, G.,	Identifying Women at High Risk for Ovarian Cancer Using Routine Lab Results	Addressing health disparities based on gender	Clin. Benefits – Diagnostic Procedures (potential)	UND
10. Viswanathan, S.	Assessing the Link between TP-R and Obesity-associated Insulin Resistance in Humans	Obesity prevention and treatment	Clin. Benefits – Investigative Procedures (demonstrated)	UNMC
11. Warren, D.	Targeted Transcranial Magnetic Stimulation to Improve Hippocampal-dependent Declarative Memory Abilities in Older Adults	Aging and age-related cognitive impairment	Clin. Benefits – Investigative Procedures (demonstrated) Clin. Benefits – Therapeutic Procedures (potential)	UNMC
12. Xie, J.	Injectable and Expandable Nanofiber Foams for Treating Noncompressible Hemorrhage	–	Clinical Benefits – Biological Factors and Products (demonstrated) Clinical Benefits – Biomedical Technology (demonstrated) Economic Benefits – Patents and License Agreements (demonstrated)	UNMC
13. Youn, J.	Development of a Wearable Intelligent System for Elderly with fall risk	Aging and age-related cognitive impairment	Community Benefits – Consumer Software (potential)	UNO
14. Zhang, C.	Predictive Modeling and Visual Analytics of Radiotherapy on Pancreatic Cancer Treatment, Diagnosis and Prognosis	–	Clin. Benefits – Therapeutic Procedures (potential)	UNL

Health Priority; BNRH – Boys town National Research Hospital; NDSU – North Dakota State University, UNL – University of Nebraska at Lincoln; UNMC – University of Nebraska Medical Center, UNO – University of Nebraska at Omaha; UND – University of North Dakota; USD – University of South Dakota.

*Projects in our study did not address these TSBM domains:* ***Clinical Benefits*** *1) Drugs; 2) Equipment and Supplies*.

***Community Benefits****: 3) Care Quality;* ***Economic Benefits****: 4) Non-Profit or Commercial Entities, 5) Cost Effectiveness, 6) Cost Savings, 7) Societal and Financial Cost of Illness; and* ***Policy Benefits****: 8) Advisory Activities Committee Participation, 9) Expert Testimony, 10) Legislation, 11) Policies and 12) Standards.*

**Figure 1.**
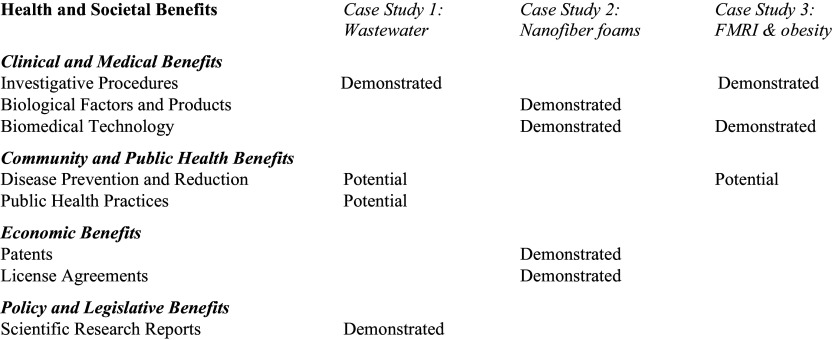
Translational Science Benefits Model Data for the Three Case Examples.

Table 1 also shows how pilot-funded projects related to benefits in the four domains of the TSMB [3]. We used a conservative approach to distinguish between demonstrated benefits (outcomes observed or verifiable) and potential benefits (future outcomes expected with least moderate confidence) [3]. Realized benefits for translational research projects in our study included Clinical and Medical Benefits (5), Economic Benefits (2), and Policy and Legislative Benefits (1) (see Figure 1).

### Subsequent Funding

To evaluate the efficiency of the investment made in Pilot awards, we used return on investment (ROI), calculated as benefit (return) of an investment divided by the cost of the investment [5]. By early 2024, 9 of 14 participants had been awarded extramural funding relating to their Pilot awards. Subsequent awards totaled $8,982,331; a return on investment of $15.23 for every $1 spent, given the total outlay of $589,936 by the GP IDeA-CTR and member institutions’ matching funding for the 14 Pilot projects. The nine projects with subsequent funding produced an ROI of $24.93 for every dollar spent, based on total GP IDeA-CTR funding of $360,443. Subsequent awards for individual projects ranged from $89,480 to $2,531,029. Funders included NIGMS and other NIH institutes, the Department of Defense, the Veterans Administration, the Robert Wood Johnson Foundation, and the Coverys Foundation.

### Lessons Learned About the Impact of GP IDeA-CTR Support

In the interviews and 2021 survey, respondents reported that Pilot funding and other support from the GP IDeA-CTR helped advance their research portfolio. Support included professional development, biostatistics services, provision of equipment, and the annual scientific meeting, which allowed investigators to showcase their work and expand their scientific network. Participants mentioned collateral benefits, including opportunities for collaboration with institutions across the network and elevating clinical and translational research within their institution, allowing more basic science-focused institutions to expand their profile in human health-related work.

Community-engaged projects reported support from local communities and organizations including facilitating recruiting and providing sites for project-related activities. Community-engaged investigators reported using fewer GP IDeA-CTR resources. This may have been in part due to five of six community-engaged projects occurring in 2017 and 2018, when community-engaged services were still being developed. Since then, the Community Engagement and Outreach Core has initiated research institutes, a community-engaged investigator interest group, and other services for investigators. This includes a practice-based research network (PBRN) which has expanded to more than 90 sites across Nebraska.

### Investigators’ Suggestions for Added Support

Some pilot awards were made soon after the initiation of the GP IDeA-CTR in 2016, and several early awardees commented on learning through “trial and error” and gaining new knowledge to address challenges, including expected ones and some they had not anticipated. Investigators of projects that were not community-engaged research reported they experienced primarily “internal” challenges, with the most common one relating to delays in the distribution of funds, which shortened the available time for projects and resulted in a few projects not expending all allocated dollars. This has been an area for focused improvement for the GP IDeA-CTR. Another challenge for clinical studies resulted from problems with electronic health record (EHR) data retrieval due to lack of IT staff at some sites. Community-engaged projects reported additional challenges that included 1) delays in obtaining clinical or community partner buy-in; 2) leadership and staff changes at community partner sites; 3) scheduling challenges that affected participant recruitment and delivery of training sessions.

In the survey and in interviews, investigators suggested a need for additional support for patient recruitment and assistance with IRB applications, particularly for behavioral and population health studies, as well as improving IRB processes for multi-institution studies. Participants also noted the GP IDeA-CTR could help clinical researchers by identifying and cultivating networks of clinicians willing to help recruit study subjects for studies and enhancing primary care physician participation in community-based projects, including studies based on EHR data. Between 2021 and 2024, these areas were addressed through expansion of the PBRN, use of the common IRB, and improved capabilities for research using EHR data.

We selected 3 projects for expanded case examples. They represent the breadth of research funded by Pilot awards, were successful in obtaining extramural funding, and highlight the versatility and breadth of the application of the TSBM to evaluate the benefits of clinical and translational research. They represent the breadth of research funded by Pilot awards, were successful in obtaining extramural funding, and highlight the versatility of the application of the TSBM to evaluate the benefits of clinical and translational research; the domains addressed are shown in the *Figure*.

*Case Example 1: Dirty Little Secrets: Wastewater Epidemiology Use to Determine Community Drug Use*

Lisa McFadden, PhD, Assistant Professor of Basic Biomedical Sciences at the University of South Dakota, received Pilot funding to analyze drugs of abuse at wastewater treatment plants in 12 US locations. Combining wastewater assessment of substances and metabolites with machine learning, the study found higher use of methamphetamine and opioids-to-methadone ratios in states west of the Mississippi.[6] It showed wastewater-based surveillance is a cost-effective public health metric for substance use; offering demonstrated benefits in two areas within Clinical and Medical Benefits and Policy and Legislative Benefits and potential benefits in two areas within Community and Public Health Benefits (see *Figure* 1). After the start of the pandemic, the knowledge and capacity developed with substance use allowed the research team to rapidly transition their wastewater analysis to study COVID-19. Dr McFadden continued work on substance use and received a subsequent NIGMS award, funded from 2020 through 2022.

*Case Example 2: Injectable and Expandable Nanofiber Foams for Treating Noncompressible Hemorrhage*

Jingwei Xie, PhD, Professor of Surgery and Research Scientist, Regenerative Medicine, UNMC, used Pilot funding to develop and test injectable, expansile nanofiber pellets that were injected to re-expand on contact with blood and achieve hemostasis for the treatment of noncompressible torso and marginally compressible junctional hemorrhage.[7] The Pilot grants offered demonstrated benefits in four areas within Clinical and Medical Benefits and Economic Benefits (see *Figure* 1). A subsequent award was funded by the Department of Defense’s Congressionally Directed Medical Research Programs from 2020 through 2023 to improve treatment of battlefield injuries by preventing exsanguination in the prehospital setting. The project resulted in a patent, licensed to Beeken Biomedical Inc., Boston, to commercialize the production of nanofiber foams.

*Case Example 3: Developing executive control, obesity risk, and behavioral health problems: A pilot fMRI study*

Timothy Nelson, Professor of Psychology at the University of Nebraska at Lincoln, received a Pilot award to conduct a functional MRI (fMRI) study to assess for risk neural vulnerability and risk factors for obesity, including food reward sensitivity and poor food regulation. Obesity is a widely prevalent condition in the United States, and the Pilot showed demonstrated benefits in two areas within Clinical and Medical Benefits and potential benefits in one area within Community and Public Health Benefits (see *Figure* 1). The subsequent study, funded by the National Institute of Diabetes and Digestive and Kidney Diseases, used longitudinal data and functional neuroimaging to assess neural vulnerability factors in young adults to inform interventions targeting individual and environmental factors to reduce obesity risk [8].

## Discussion

We chose the TSBM and an intermediate follow-up period to examine whether Pilot funding awards in subsequent funding as well as pragmatic benefits for the health of individuals and communities. Unlike some other models of impact, which rely on bibliometric data and process metrics [9,10], the TSBM focuses on the end points of research, creating a bridge between the perspectives of investigators, clinicians, policymakers, and others who stand to benefit from the outcomes of research. We demonstrated that TSBM indicators are compatible with the GP IDeA-CTR’s Health Priorities List, as both focus on the range of medical, technological, public health, and systems-based approaches to address regional health concerns. The bridge to pragmatic outcomes for clinical and translational research may also facilitate the research translation process through partnerships among investigators and communities of research end-users [11]. This may ultimately contribute to improved understanding of these partnerships, including their conceptual models, initiation processes, enablers, barriers, and outcomes [11].

Limitations of our case study include the lack of data on whether projects resulted in improved health indicators for conditions on the HPL and other outcome targets. There also is limited data on demonstrated TSBM impact for the projects in our study. Our description of challenges is based on a convenience sample and may not represent all challenges experienced by Pilot-funded clinical and translational research projects. There also is a potential for “reporting bias,” as investigators with successful projects may have been more likely to respond to our follow-up survey.

## Conclusion

We used an efficient and low-burden approach to evaluate the impact of Pilot-funded clinical and translational research projects, showing benefits in the four domains of the TSBM as endpoints of successful research translation. We confirmed that Pilot funding led to subsequent extramural funding for the majority of investigators in our study. While many TSBM outcomes were prospective (potential) at the time we conducted this study, projects may ultimately create demonstrated benefits for patients and populations. The GP IDeA-CTR will continue to follow the work of its awardees using pragmatic metrics that are of value to investigators, member institutions, funders, and the public who all stand to benefit from this work.
